# Clinical outcomes of long-term inhaled combination therapies in patients with bronchiectasis and airflow obstruction

**DOI:** 10.1186/s12890-024-02867-4

**Published:** 2024-01-23

**Authors:** Hyo Jin Lee, Jung-Kyu Lee, Tae Yeon Park, Eun Young Heo, Deog Kyeom Kim, Hyun Woo Lee

**Affiliations:** 1 Division of Respiratory and Critical Care, Department of Internal Medicine, Seoul Metropolitan Government-Seoul National University Boramae Medical Center, Seoul, South Korea; 2 Division of Pulmonary and Critical Care Medicine, Department of Internal Medicine, Seoul Metropolitan Government-Seoul National University Boramae Medical Center, 20, Boramae-ro 5-gil, Dongjak-gu, Seoul, 07061 South Korea

**Keywords:** Bronchiectasis, COPD, Inhaled corticosteroid, Bronchodilator agent, FEV_1_, Exacerbation

## Abstract

**Background and objectives:**

Few studies have reported which inhaled combination therapy, either bronchodilators and/or inhaled corticosteroids (ICSs), is beneficial in patients with bronchiectasis and airflow obstruction. Our study compared the efficacy and safety among different inhaled combination therapies in patients with bronchiectasis and airflow obstruction.

**Methods:**

Our retrospective study analyzed the patients with forced expiratory volume in 1 s (FEV_1_)/forced vital capacity < 0.7 and radiologically confirmed bronchiectasis in chest computed tomography between January 2005 and December 2021. The eligible patients underwent baseline and follow-up spirometric assessments. The primary endpoint was the development of a moderate-to-severe exacerbation. The secondary endpoints were the change in the annual FEV_1_ and the adverse events. Subgroup analyses were performed according to the blood eosinophil count (BEC).

**Results:**

Among 179 patients, the ICS/long-acting beta-agonist (LABA)/long-acting muscarinic antagonist (LAMA), ICS/LABA, and LABA/LAMA groups were comprised of 58 (32.4%), 52 (29.1%), and 69 (38.5%) patients, respectively. ICS/LABA/LAMA group had a higher severity of bronchiectasis and airflow obstruction, than other groups. In the subgroup with BEC ≥ 300/uL, the risk of moderate-to-severe exacerbation was lower in the ICS/LABA/LAMA group (adjusted HR = 0.137 [95% CI = 0.034–0.553]) and the ICS/LABA group (adjusted HR = 0.196 [95% CI = 0.045–0.861]) compared with the LABA/LAMA group. The annual FEV_1_ decline rate was significantly worsened in the ICS/LABA group compared to the LABA/LAMA group (adjusted β-coefficient=-197 [95% CI=-307–-87]) in the subgroup with BEC < 200/uL.

**Conclusion:**

In patients with bronchiectasis and airflow obstruction, the use of ICS/LABA/LAMA and ICS/LABA demonstrated a reduced risk of exacerbation compared to LABA/LAMA therapy in those with BEC ≥ 300/uL. Conversely, for those with BEC < 200/uL, the use of ICS/LABA was associated with an accelerated decline in FEV_1_ in comparison to LABA/LAMA therapy. Further assessment of BEC is necessary as a potential biomarker for the use of ICS in patients with bronchiectasis and airflow obstruction.

**Supplementary Information:**

The online version contains supplementary material available at 10.1186/s12890-024-02867-4.

## Abstract (pa)

ICS/LABA/LAMA and ICS/LABA may be more beneficial for reducing moderate-to-severe exacerbations than LABA/LAMA in patients with eosinophilic bronchiectasis and airflow obstruction.

## Introduction

Bronchiectasis is a chronic airway disease characterised by neutrophilic bronchial inflammation and is commonly reported in the patients with airflow obstruction including asthma [1] or chronic obstructive pulmonary disease (COPD) [2]. As bronchiectasis is diagnosed based on structural abnormality in radiologic evaluation while COPD is diagnosed based on physiologic abnormality in spirometric evaluation, both diagnoses can be fulfilled in a patient with bronchiectasis-COPD overlap (BCO) [3]. With increasing use of screening chest computed tomography (CT) in the patients who ever smoked, BCO has been increasingly documented and the clinical relevance of BCO has been emerging. The patients with bronchiectasis and airflow obstruction had a higher risk of acute exacerbations [4] and mortality [5] than those with bronchiectasis alone. In addition, COPD patients with bronchiectasis had more symptoms, a higher bacterial burden, and a higher risk of acute exacerbation [2].

The effective treatment for bronchiectasis and airflow obstruction has not been sufficiently evaluated. Long-acting beta-2 agonists (LABAs), long-acting muscarinic antagonists (LAMAs), and inhaled corticosteroids (ICSs) have been important drugs for treating COPD. However, their effectiveness in bronchiectasis is less evident [6]. In a randomized controlled trial (RCT), ICS/LABA improved the symptoms and quality of life more than ICS in patients with bronchiectasis [7]. The lung function was numerically more improved with LABA/LAMA than with LABA or LAMA in patients with bronchiectasis [8]. In patients with bronchiectasis, the use of ICS is cautiously considered due to concerns regarding their potential impact on respiratory infections and long-term safety. However, it is still unclear whether inhaled combination therapy with ICS can be beneficial in patients with bronchiectasis and airflow obstruction, especially who had eosinophilia.

The present study aimed to compare the development of acute exacerbation, the change in lung function, and adverse events among patients with bronchiectasis and airflow obstruction treated with ICS/LABA/LAMA, ICS/LABA, and LABA/LAMA.

## Materials and methods

The present study followed the guidance presented by the Strengthening the Reporting of Observational Studies in Epidemiology (STROBE) statement [9].

### Study design and participants

This retrospective study assessed all patients aged ≥ 18 years with forced expiratory volume in 1 s (FEV_1_)/forced vital capacity (FVC) < 70% [10] and radiologically confirmed bronchiectasis in chest CT [11] between January 2005 and December 2021 in the Seoul Metropolitan Government–Seoul National University (SMG-SNU) Boramae Medical Center. We included the patients with bronchiectasis and airflow obstruction who underwent baseline and two or more annual follow-up spirometric assessments, experienced an acute exacerbation event during the past year, and were adherent to inhaled combination therapy for at least 6 months. We observed the longest follow-up period during which treatment adherence was appropriate for individual patients. Treatment adherence was assessed by whether inhaled drugs were regularly prescribed. The patients received initial training for the use of the inhaler devices at the first prescription and additional trainings by checking the patient’s technique for the prescribed inhaler devices during follow-up. The eligible patients were classified into 3 groups: ICS/LABA/LAMA, ICS/LABA, and LABA/LAMA groups.

### Pulmonary function test

The highest measured FVC and FEV_1_ among three or more tests with acceptable curves were used. The absolute values of FVC and FEV_1_ were obtained, and the percentage of predicted values for FEV_1_ and FVC were calculated from the Morris equations [12]. Airflow limitation was defined as FEV_1_/FVC < 0.7 by spirometric evaluation based on the American Thoracic Society/European Respiratory Society guidelines [13]. The positive bronchodilator response (BDR) at baseline was defined as a postbronchodilator increase in FEV_1_ and/or FVC of at least 12% and 200 mL from baseline values at 15 min after inhalation of 400 µg of salbutamol [14]. Spirometry was conducted by a well-trained technician using a same Vmax series Sensor Medics 2130 automatically computerized spirometry system (SensorMedics) according to official statements of the American Thoracic Society and European Respiratory Society in 2019 [15].

### Variables

Baseline information, including age, sex, body mass index (BMI), smoking history, disease severity, previous exacerbation history, bacterial colonization, comorbidities, and treatment duration, was obtained. The history of exacerbations was assessed based on the electronic medical records of the patients. The severity of bronchiectasis was assessed with the Bronchiectasis Severity Index (BSI) and FACED score [16]. Clinical features, including etiology, respiratory symptoms, adjuvant treatments, laboratory tests, spirometric examination, predominant morphology, and number of lobes that were involved, were collected. The basic morphologic types of bronchiectasis (cylindrical, varicose, and cystic) and the involved lobes on chest CT were evaluated by two pulmonologists.

### Outcomes

The primary endpoint was to compare the risk of moderate-to-severe exacerbation among the ICS/LABA/LAMA, ICS/LABA, and LABA/LAMA groups. A moderate exacerbation was defined as an exacerbation leading to treatment with antibiotics or systemic glucocorticoids. A severe exacerbation was one resulting in hospitalization or death [6, 17, 18]. Secondary endpoints were to compare the annual FEV_1_ change (mL/yr) and the development of adverse events, including pneumonia, MACE, and mortality. The risk of moderate-to-severe exacerbation and the annual FEV_1_ change were evaluated according to blood eosinophil count (BEC). The baseline measurement of BEC was obtained during the stable phase of the patient’s disease severity.

### Statistical analysis

Data are presented as the mean with standard deviation or the median with interquartile range (IQR) for continuous variables and numbers with percentage for categorical variables. Analysis of variance (ANOVA) test was used to test independent samples of continuous, normally distributed data, while the Wilcoxon rank-sum test was used to examine continuous, skewed data. The chi-square test or Fisher’s exact test was used to analyze categorical data. Kaplan–Meier curves and log-rank tests were performed to compare the time to first moderate-to-severe exacerbation among the ICS/LABA/LAMA, ICS/LABA, and LABA/LAMA groups. We conducted univariable Cox regression analyses for moderate-to-severe exacerbation among the ICS/LABA/LAMA, ICS/LABA, and LABA/LAMA groups. For multivariable Cox regression analysis, clinically relevant variables were selected through backward elimination method. Clinically relevant variables included age, sex, BMI, current smoking status, mMRC grade, BSI score, FACED score, history of previous moderate-to-severe exacerbation, number of exacerbations in the last 12 months, lung cancer, BEC > 300/uL, high-sensitivity C-reactive protein, baseline FEV_1_, baseline FEV_1_/FVC ratio, positive bronchodilator response, chronic infection with *Pseudomonas aeruginosa*, and radiologic severity. A linear mixed model was used to estimate the effect of the clinical factors contributing to the annual FEV_1_ change (mL/yr). For multivariable linear mixed model, clinically relevant variables were selected through backward elimination method. *P* < 0.05 was considered as statistical significance. A variance inflation factor (VIF) > 4.0 was considered as significant multicollinearity. Even though statistical multicollinearity was not confirmed, but high intercorrelation was clinically suspected (e.g. severity score systems and their components), one of the correlated variables was excluded from the multivariable model. We used R statistical software, version 3.6.3 (R Core Team [2020], Vienna, Austria), for statistical analyses.

### Ethics

Our study was conducted by following the principles of the Declaration of Helsinki. The institutional review board of the SMG–SNU Boramae Medical Center approved this study and waived the requirement for informed consent (IRB No. 10-2020-099).

## Results

**Fig. 1:**
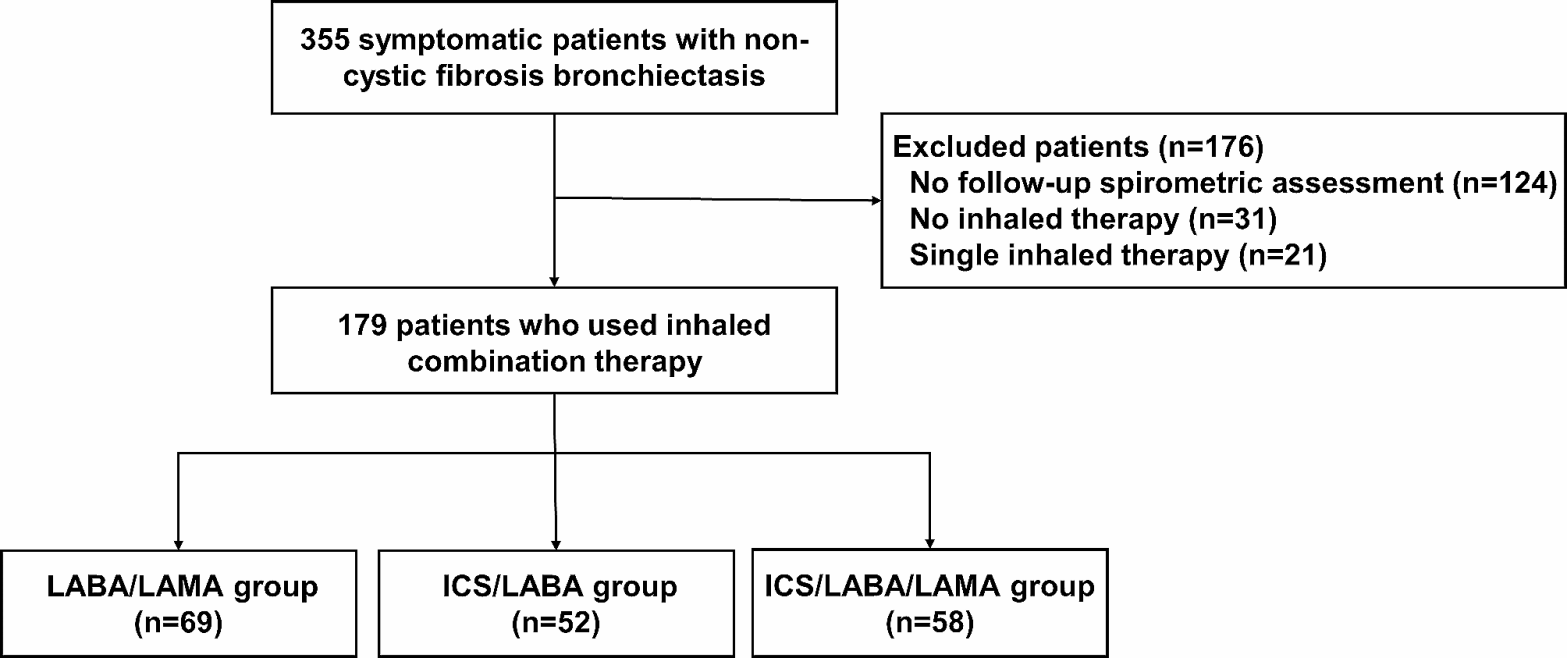
Flowchart of patient inclusion and exclusion

Among a total of 355 patients with bronchiectasis and airflow obstruction, 176 patients were excluded because 124 did not undergo at least 2 annual spirometric assessments, 31 were not treated with any inhaled therapy, and 21 were treated with single inhaled therapy. None of the included patients were diagnosed as cystic fibrosis or alpha-1 antitrypsin deficiency. The eligible 179 patients were assigned to the ICS/LABA/LAMA group (*n* = 58), the ICS/LABA group (*n* = 52), and the LABA/LAMA group (*n* = 69) (Fig. 1). They underwent a median of 4 (IQR = 3–5) annual spirometric assessments, and their median annual FEV_1_ change was − 89 (IQR= -364–291) mL/yr. The median follow-up duration was 40 [IQR = 23–62] months.

### Baseline characteristics and clinical features

The baseline characteristics of the included patients are described in Table 1. There were significant differences in sex, BMI, smoking history, disease severity, and previous exacerbation history between the three groups. The ICS/LABA/LAMA group showed a lower BMI, a higher likelihood of smoking, a greater severity of bronchiectasis, and a history of more severe previous exacerbation event compared to both the LABA/LAMA and ICS/LABA groups. Considering the FACED score, the LABA/LAMA group had a higher severity of bronchiectasis than the ICS/LABA group. The proportion of overall detected bacteria that colonized the lungs was 31% and was comparable between the three groups. The treatment duration with inhaled combination therapy was significantly longer in the ICS/LABA/LAMA and ICS/LABA groups than in the LABA/LAMA group. The results of post-hoc analysis are described in **Supplementary information **1 and 2.

**Table 1:** Baseline characteristics of patients with bronchiectasis and airflow obstruction treated with inhaled combination therapies

	ICS/LABA/LAMA (*n* = 58)	ICS/LABA (*n* = 52)	LABA/LAMA (*n* = 69)	P-value
**Age, mean (SD)**	61.05 (10.35)	62.60 (12.19)	63.81 (11.34)	0.392
**Female, n (%)**	7 (12.1)	20 (38.5)	15 (21.7)	0.004
**BMI, mean (SD)**	20.44 (4.06)	22.86 (4.42)	21.86 (3.66)	0.011
**Smoking history**				
Never smoker, n (%)	10 (17.2)	20 (38.5)	25 (36.2)	0.025
Ex-smoker, n (%)	25 (43.1)	18 (34.6)	29 (42.0)	0.615
Current smoker, n (%)	23 (39.7)	14 (26.9)	15 (21.7)	0.079
Pack years, median (IQR)	30 (22–38)	15 (9–21)	20 (14–26)	0.002
**Disease severity**				
mMRC score, mean (SD)	2.09 (0.82)	1.63 (0.66)	1.75 (0.81)	0.007
BSI score, mean (SD)	7.91 (3.75)	5.29 (2.80)	6.06 (3.25)	< 0.001
FACED score, mean (SD)	2.66 (1.66)	1.63 (1.69)	2.29 (1.48)	0.004
**Previous moderate or severe exacerbation history, n (%)**	24 (43.6)	14 (29.2)	14 (20.3)	0.019
**Bacterial colonizer, n (%)**	21 (36.2)	14 (26.9)	21 (30.4)	0.566
Pseudomonas colonizer, n (%)	7 (12.1)	2 (3.8)	6 (9.2)	0.298
**Comorbidity**				
Hypertension, n (%)	24 (41.4)	24 (46.2)	33 (47.8)	0.759
Diabetes mellitus, n (%)	11 (19.0)	17 (32.7)	20 (29.0)	0.234
Chronic kidney disease, n (%)	6 (10.3)	3 (5.8)	6 (8.7)	0.683
Chronic liver disease, n (%)	6 (10.3)	7 (13.5)	10 (14.5)	0.775
Cerebrovascular disease, n (%)	6 (10.3)	6 (11.5)	7 (10.1)	0.967
Cardiovascular disease, n (%)	9 (15.5)	13 (25.0)	14 (20.3)	0.464
Lung cancer, n (%)	7 (12.1)	4 (7.7)	15 (21.7)	0.077
Malignancy other than lung cancer, n (%)	10 (17.2)	12 (23.1)	9 (13.0)	0.353
**Duration of inhaled combination therapy, month, mean (SD)**	62.81 (39.15)	55.96 (44.83)	33.34 (17.63)	< 0.001

**Note**: Data presented as n (%) for categorical variables or mean (SD) or median (IQR) for numerical variables

**Abbreviations**: ICS, inhaled corticosteroid; LABA, long-acting β2-agonist; LAMA, long-acting muscarinic antagonist; BMI, body mass index; mMRC, Modified Medical Research Council dyspnea scale; BSI, Bronchiectasis Severity Index; FACED, forced expiratory volume in 1 s, age, chronic infection with Pseudomonas, radiological extension and dyspnea; COPD, chronic obstructive pulmonary disease; ACO, Asthma and COPD overlap; NTM?PD, nontuberculous mycobacteria pulmonary disease

Regarding the clinical features, we found no difference in the etiology of bronchiectasis among the three groups (Table 2). Non-purulent sputum was more abundant, and mucolytics, including N-acetylcysteine and erdosteine, were more commonly used in the ICS/LABA/LAMA group than in the LABA/LAMA and ICS/LABA groups. In addition, the proportion of patients requiring long–term oxygen therapy was significantly higher in the ICS/LABA/LAMA group. ICS/LABA/LAMA and ICS/LABA groups showed a higher BEC compared to LABA/LAMA group. At the baseline spirometric examination, FEV_1_ (%) and FEV_1_/FVC (%) were significantly lower in the ICS/LABA/LAMA and LABA/LAMA groups than in the ICS/LABA group. The ICS/LABA group had a higher FVC (%) than the LABA/LAMA group and a higher DLCO/VA (%) than the ICS/LABA/LAMA group. The BDR positivity was significantly lower in LABA/LAMA than in ICS/LABA and ICS/LABA/LAMA. There was no difference in the morphologic features or the involved pulmonary lobes of bronchiectasis in chest CT among the three groups.

**Table 2:** Clinical features of patients with bronchiectasis and airflow obstruction treated with inhaled combination therapies

	ICS/LABA/LAMA (*n* = 58)	ICS/LABA (*n* = 52)	LABA/LAMA (*n* = 69)	P-value
**Etiology of bronchiectasis**				
Post-infectious, n (%)	28 (48.3)	14 (26.9)	29 (42.0)	0.064
Idiopathic, n (%)	16 (27.6)	18 (34.6)	24 (34.8)	0.635
Chronic airway disease, n (%)	9 (15.5)	10 (19.2)	8 (11.6)	0.506
ABPA, n (%)	2 (3.4)	5 (9.6)	3 (4.3)	0.316
GERD, n (%)	2 (3.4)	4 (7.7)	3 (4.3)	0.565
Connective tissue disease, n (%)	0	1 (1.9)	1 (1.4)	0.598
Immunosuppression, n (%)	1 (1.7)	0 (0.0)	1 (1.4)	0.654
**Respiratory symptoms**				
Cough, n (%)	25 (43.1)	23 (44.2)	25 (36.2)	0.614
Non-purulent sputum, n (%)	20 (34.5)	8 (15.4)	7 (10.1)	0.002
Purulent sputum, n (%)	12 (20.7)	9 (17.3)	17 (24.6)	0.616
Hemoptysis, n (%)	11 (19.0)	9 (17.3)	18 (26.1)	0.443
Chest discomfort, n (%)	2 (3.4)	1 (1.9)	1 (1.4)	0.738
Dyspnea, n (%)	45 (77.6)	34 (65.4)	45 (65.2)	0.248
**Adjuvant treatments**				
N-acetylcystein, n (%)	19 (32.8)	9 (17.3)	11 (15.9)	0.048
Ambroxol, n (%)	19 (32.8)	11 (21.2)	20 (29.0)	0.388
Erdosteine, n (%)	39 (67.2)	26 (50.0)	31 (44.9)	0.035
Bronchial artery embolization history, n (%)	9 (15.5)	4 (7.7)	14 (20.3)	0.158
Long–term oxygen therapy, n (%)	47 (81.0)	33 (64.7)	41 (59.4)	0.029
**Laboratory tests**				
White blood cell, 1000/uL mean (SD)	9.54 (13.03)	7.65 (2.39)	8.03 (2.87)	0.391
Hemoglobin, g/dl, mean (SD)	14.45 (3.69)	13.46 (1.48)	13.02 (1.74)	0.006
Platelet, 1000/uL, mean (SD)	248 (79)	266 (87)	248 (93)	0.459
Blood eosinophil count, /uL, mean (SD)	408 (223)	467 (355)	239 (223)	< 0.001
Blood eosinophil count, n (%)				
< 150/uL	0	0	28 (41.2)	< 0.001
150–299/uL	20 (34.5)	22 (42.3)	23 (33.8)	0.529
≥ 300/uL	38 (65.5)	30 (57.7)	17 (25.0)	< 0.001
hs-CRP, median (IQR)	0.78 (0–2.05)	0.35 (0–0.70)	1.56 (0.89–2.23)	0.001
**Spirometric examination**				
FVC, L, mean (SD)	2.79 (0.72)	2.63 (0.98)	2.43 (0.69)	0.035
FVC, %, mean (SD)	76.38 (17.34)	79.75 (20.02)	70.80 (16.84)	0.023
FEV_1_, L, mean (SD)	1.22 (0.43)	1.44 (0.61)	1.19 (0.44)	0.016
FEV_1_, %, mean (SD)	47.33 (15.07)	62.87 (22.85)	49.86 (15.56)	< 0.001
FEV_1_/FVC, %, mean (SD)	44.13 (12.32)	56.02 (13.37)	49.66 (12.96)	< 0.001
DL_CO_, L, mean (SD)	10.73 (4.88)	13.10 (5.59)	10.89 (5.16)	0.046
DL_CO_, %, mean (SD)	63.54 (22.29)	75.23 (27.99)	66.89 (27.90)	0.083
DL_CO_/VA, L, mean (SD)	2.92 (1.13)	3.54 (1.15)	3.08 (1.26)	0.031
DL_CO_/VA, %, mean (SD)	76.85 (27.87)	93.25 (28.10)	82.11 (31.59)	0.022
BDR positivity, n (%)	15 (25.9)	16 (30.8)	6 (8.8)	0.007
**Predominant morphology in CT**				
Cylindrical, n (%)	27 (46.6)	22 (42.3)	22 (31.9)	0.218
Varicose, n (%)	17 (29.3)	18 (34.6)	29 (42.0)	0.323
Cystic, n (%)	14 (24.1)	12 (23.1)	18 (26.1)	0.926
**Total number of lobe involvement in CT, mean (SD)**	2.64 (1.53)	2.29 (1.42)	2.75 (1.58)	0.238

**Note**: Data presented as n (%) for categorical variables or mean (SD) or median (IQR) for numerical variables

**Abbreviations**: ABPA, allergic bronchopulmonary aspergillosis; BAE, bronchial artery embolization; BDR, bronchodilator response; CT, computed tomography; DL_CO_, diffusing capacity of the lungs for carbon monoxide; FVC, forced vital capacity; FEV1, forced expiratory volume in 1 second; GERD, gastroesophageal reflux disease; ICS, inhaled corticosteroid; LABA, long-acting β2-agonist; LAMA, long-acting muscarinic antagonist; SD, standard deviation; SE, standard error; VA, alveolar volume

### Exacerbation

**Fig. 2:**
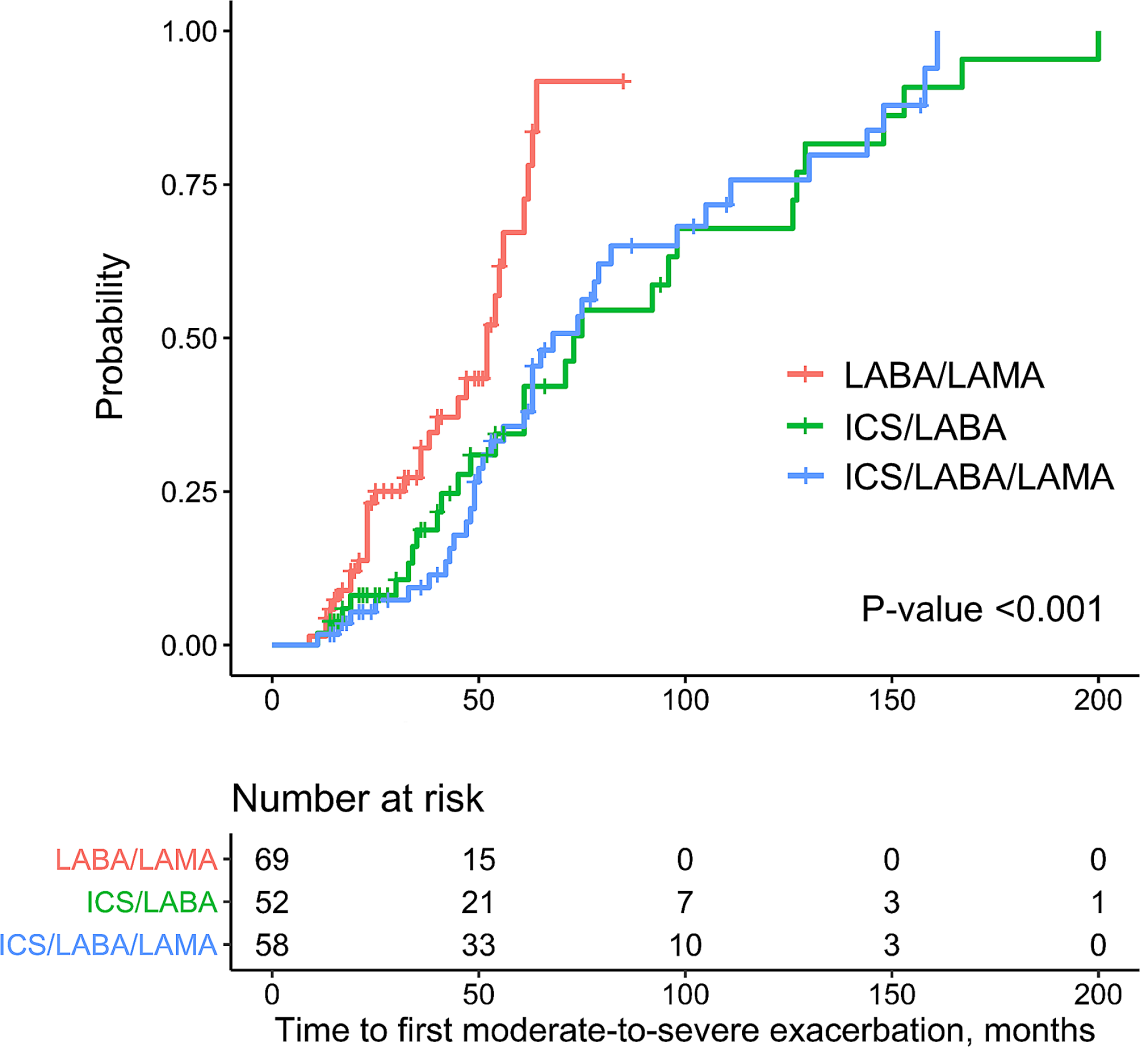
Kaplan-Meier curves for the time to first moderate to severe bronchiectasis exacerbation according to the inhaled therapy group

The number of moderate-to-severe exacerbation events was not significantly different among the three groups. However, the time to the first event of a moderate-to-severe exacerbation was significantly shorter in the LABA/LAMA group than in the ICS/LABA and ICS/LABA/LAMA groups (log-rank test, P-value < 0.001, Fig. 2). In the univariable Cox regression model, older age, a higher grade of mMRC, a higher score of BSI, a higher score of FACED, previous history of moderate-to-severe exacerbation, and a higher number of exacerbations in previous year were related to an increased hazard of a moderate–to–severe exacerbation in the patients with bronchiectasis and airflow obstruction (Table 3). However, in the multivariable Cox regression analysis, the hazard of moderate-to-severe exacerbation in the ICS/LABA and ICS/LABA/LAMA groups was not significantly different from the LABA/LAMA groups (ICS/LABA vs. LABA/LAMA, adjusted HR = 0.491 [95% CI = 0.191–1.263], P-value = 0.140; ICS/LABA/LAMA vs. LABA/LAMA, adjusted HR = 0.692 [95% CI = 0.293–1.638], P-value = 0.403) The adjusted hazard for moderate-to-severe exacerbation was not statistically different between the ICS/LABA and ICS/LABA/LAMA groups.

**Table 3:** Hazard ratio of moderate-to-severe exacerbation in patients with bronchiectasis and airflow obstruction treated with inhaled combination therapies

	Univariable Cox regression model		Multivariable Cox regression model	
	HR (95% CI)	P-value	HR (95% CI)	P-value
**Age**	1.030 (1.009–1.052)	0.006	1.035 (1.012–1.059)	0.003
**Female**	0.898 (0.563–1.430)	0.649		
**BMI**	1.019 (0.968–1.072)	0.476		
**Current smoker**	1.276 (0.812–2.007)	0.291	2.041 (1.209–3.447)	0.007
**mMRC grade**	1.408 (1.081–1.834)	0.011	1.315 (1.012–1.7309)	0.030
**BSI score**	1.076 (1.019–1.137)	0.009		
**FACED score**	1.140 (1.015–1.280)	0.027		
**Previous moderate or severe exacerbation history**	1.546 (1.005–2.379)	0.047		
**Number of exacerbations in previous year**	2.539 (2.002–3.220)	< 0.001	1.617 (1.017–2.571)	< 0.037
**Lung cancer**	1.309 (0.705–2.430)	0.394		
**Blood eosinophil count > 300/uL**	0.559 (0.369–0.845)	0.006	0.583 (0.350–0.971)	0.038
**hs-CRP**	1.001 (0.943–1.064)	0.962		
**Baseline FEV**_**1**_ **(L)**	0.614 (0.364–1.037)	0.068		
**Baseline FEV**_**1**_ **/FVC (%)**	0.997 (0.981–1.013)	0.703		
**Baseline DL**_**CO**_, **(%)**	0.852 (0.696–1.043)	0.120	0.988 (0.977–0.998)	0.024
**Positive bronchodilator response**	0.767 (0.475–1.238)	0.277		
***Colonization with Pseudomonasaeruginosa***	1.710 (0.746–3.918)	0.205		
**Total number of lobe involvement in CT**	0.981 (0.851–1.130)	0.788		
**Inhaled combination therapy (ref: LABA/LAMA)**				
ICS/LABA	0.369 (0.207–0.658)	< 0.001	0.491 (0.191–1.263)	0.140
ICS/LABA/LAMA	0.413 (0.244–0.699)	< 0.001	0.692 (0.293–1.638)	0.403

**Note**: Data were analyzed with univariable and multivariable cox regression models and are presented as adjusted hazard ratio (95% confidence interval). BSI and FACED were omitted from the multivariable analysis due to concerns of collinearity with other clinical variables.

**Abbreviations**: BMI, body mass index; BSI, Bronchiectasis Severity Index; CI, confidence interval; COPD, chronic obstructive pulmonary disease; CT, computed tomography; DL_CO_, diffusing capacity of the lungs for carbon monoxide; FACED, forced expiratory volume in 1 s, age, chronic infection with Pseudomonas, radiological extension and dyspnea; FEV_1_, forced expiratory volume in 1 second; FVC, forced vital capacity; HR, hazard ratio; hs-CRP, high-sensitivity C-reactive protein; ICS, inhaled corticosteroid; LABA, long-acting β2-agonist; LAMA, long-acting muscarinic antagonist; mMRC, Modified Medical Research Council dyspnea scale

### Lung function decline rate

In the multivariable linear mixed effect model, elderly, female, a lower BMI, current smoker, a higher grade of mMRC, a lower baseline FEV_1_, and previous history of moderate-to-severe exacerbation were related with accelerated annual FEV_1_ decline rate **(**Table 4**)**. There was no significant difference in annual FEV_1_ decline rate among the ICS/LABA/LAMA, ICS/LABA, and LABA/LAMA groups.

**Table 4:** Effect of clinical factors contributing to the annual FEV_1_ change (mL/yr)

	Adjusted β-coefficient (95% CI)	P-value
**Age**	-4.18 (-6.19–-2.17)	< 0.001
**Female**	-121.15 (-172.84–-69.47)	< 0.001
**BMI**	11.29 (5.99–16.59)	< 0.001
**Current smoker**	-76.61 (-123.54–-29.69)	0.001
**mMRC grade**	-34.12 (-62.31–-5.93)	0.018
**Baseline FEV**_**1**_ **(100 mL)**	75.25 (70.17–80.32)	< 0.001
**Previous moderate or severe exacerbation history**	-50.51 (-97.51–-3.50)	0.036
**Inhaled therapy (ref: LABA/LAMA)**		
ICS/LABA	-49.73 (-105.47–6.01)	0.081
ICS/LABA/LAMA	48.83 (-1.94–99.59)	0.060

**Note**: Data were analyzed with mixed linear regression and are presented as linear regression coefficient and standard error. BSI and FACED were omitted from the multivariable analysis due to concerns of collinearity with other clinical variables.

**Abbreviations**: BMI, body mass index; FEV_1_, forced expiratory volume in 1 second; LABA, long-acting β2-agonist; LAMA, long-acting muscarinic antagonist

### Subgroup analysis according to BEC

The ICS/LABA/LAMA group had a lower risk of moderate-to-severe exacerbation in subgroup with BEC ≥ 300/uL (adjusted HR = 0.137 [95% CI = 0.034–0.553], P-value = 0.005) than LABA/LAMA group (Table 5). In the subgroup with BEC ≥ 300/uL, annual FEV_1_ decline rate was numerically more attenuated without statistical significance in the ICS/LABA/LAMA group compared to LABA/LAMA group (adjusted β-coefficient = 246.45 [95% CI=-63.80–556.70]], P-value = 0.128) and in the subgroup with BEC = 150–299/uL (adjusted β-coefficient = 191.80 [95% CI=-39.03–422.64], P-value = 0.123).

**Table 5:** Acute exacerbation and annual FEV_1_ change according to the blood eosinophil count

	Moderate-to-severe exacerbation			Annual FEV_1_ change, mL/yr		
	adjusted HR^a^ (95% CI)	P-value			Adjusted β-coefficient^b^ (95% CI)	P-value
Blood eosinophil count, ≥ 300/uL						
ICS/LABA (compared with LABA/LAMA)	0.196 (0.045–0.861)		0.031	-137.06 (-428.66–154.53)		0.361
ICS/LABA/LAMA (compared with LABA/LAMA)	0.137 (0.034–0.553)		0.005	246.45 (-63.80–556.70)		0.128
**Blood eosinophil count, 200–299/uL**						
ICS/LABA (compared with LABA/LAMA)	0.944 (0.175–5.101)		0.947	-42.72 (-398.09–312.65)		0.817
ICS/LABA/LAMA (compared with LABA/LAMA)	0.655 (0.098–4.388)		0.663	191.80 (-39.03–422.64)		0.123
**Blood eosinophil count, < 200/uL**						
ICS/LABA (compared with LABA/LAMA)	1.918 (0.465–7.908)		0.368	-197.18 (-307.04–-87.32)		< 0.001
ICS/LABA/LAMA (compared with LABA/LAMA)	1.467 (0.462–4.658)		0.515	9.63 (-115.23–134.49)		0.880

**Note**: Data were analyzed with mixed linear regression and are presented as linear regression coefficient and standard error

^a^ Adjusted hazard ratio was estimated using covariables including age, current smoker, mMRC grade, baseline FEV_1_, and number of exacerbations in previous year.

^b^ Adjusted β-coefficient was estimated using covariables including age, sex, BMI, current smoker, mMRC grade, baseline FEV_1_, and previous history of moderate or severe exacerbation.

ICS/LABA group showed a lower risk of moderate-to-severe exacerbation (adjusted HR = 0.196 [95% CI = 0.045–0.861], P-value = 0.005) compared to LABA/LAMA group in the subgroup with BEC ≥ 300/uL (Table 5). In addition, annual FEV_1_ decline rate was more accelerated in the ICS/LABA group compared to LABA/LAMA group in the subgroup with BEC < 200/uL (adjusted β-coefficient=-197.18 [95% CI=-307.04–-87.32], P-value < 0.001).

### Adverse events

There was no significant difference in the development of pneumonia between the ICS/LABA/LAMA (*n* = 40, 69.0%), ICS/LABA (*n* = 32, 61.5%), and LABA/LAMA (*n* = 44, 62.9%) groups. MACE was similarly reported among the ICS/LABA/LAMA (*n* = 8, 13.8%), ICS/LABA (*n* = 11, 21.2%), and LABA/LAMA (*n* = 14, 20.0%) groups. We found no difference in mortality events among the ICS/LABA/LAMA (*n* = 7, 12.1%), ICS/LABA (*n* = 2, 3.8%), and LABA/LAMA (*n* = 7, 10.0%) groups.

## Discussion

Our longitudinal observational study compared the efficacy and safety of ICS/LABA/LAMA, ICS/LABA, and LABA/LAMA treatments in the patients with bronchiectasis and airflow obstruction. In the baseline clinical features, more symptoms, greater severity of bronchiectasis, and a history of more frequent exacerbations were found in the ICS/LABA/LAMA group than in the LABA/LAMA or ICS/LABA groups. In addition, the ICS/LABA/LAMA group had a lower baseline FEV_1_ and FEV_1_/FVC than the ICS/LABA group. Despite these differences, the ICS/LABA/LAMA group did not show a significant difference in the adjusted HR for moderate-to-severe exacerbation compared to the ICS/LABA group and LABA/LAMA group. However, the beneficial effect of ICS/LABA/LAMA and ICS/LABA in reducing moderate-to-severe exacerbation was observed in patients with BEC ≥ 300/uL. There was no significant difference in reducing the annual FEV_1_ decline rate among the ICS/LABA/LAMA, ICS/LABA, and LABA/LAMA groups. However, ICS/LABA was associated with an accelerated FEV_1_ decline in those with BEC ≥ 300/uL compared to the LABA/LAMA group. We found no difference in the incidence of pneumonia, MACE, or death among the three groups. Combination therapy with ICS may have benefits in preventing clinical deterioration in the patients with bronchiectasis and airway obstruction in the presence of high BEC.

There have been several efforts to determine the beneficial effects of inhaled therapies in bronchiectasis. In a prospective study with 77 patients, budesonide group showed numerically less exacerbations and more improvement of FEV_1_ without statistical significance [19]. Another prospective study analyzed the effect of inhaled beclomethasone diproprionate for 6 weeks in 20 patients with bronchiectasis and revealed a significant improvement in the FEV_1_ [20]. In a clinical trial, inhaled combination therapy with medium-dose budesonide and formoterol was compared for a year with high-dose budesonide in patients with bronchiectasis [7]. In this study, patients with medium-dose budesonide and formoterol had a better improvement in symptoms but did not show improvements in their lung function or in reducing acute exacerbation [7]. However, those results from previous studies have been interpreted limitedly in that the number of study participants was small (fewer than 100) and the treatment duration was as short as a year or less. Compared with previous studies, we included the patients with bronchiectasis who had airflow limitation (FEV_1_/FVC < 0.7) and followed them up for a longer period (more than 3 years). We could find a potential role of ICS for the patients with eosinophilic bronchiectasis and airflow obstruction, who had BEC ≥ 300/uL. More studies are needed to clarify the potential benefits of using inhaled combination therapy with ICS among patients with eosinophilic bronchiectasis and airflow obstruction.

In our study, the ICS/LABA/LAMA and ICS/LABA groups were associated with a lower exacerbation risk than the LABA/LAMA group when the bacterial load including *Pseudomonas aeruginosa (PA)* was 31% and comparable between the three groups. The prevalence of *PA* infection or colonizer in non-cystic bronchiectasis has been reported from 9 to 34% in several studies [22–28]. In individuals diagnosed with non-cystic fibrosis bronchiectasis, infection with *PA* is correlated with heightened sputum production, diminished lung function, and a deceleration of respiratory ciliary beat in vivo [24, 27, 29]. Interestingly, a retrospective study showed a reduction in the exacerbation frequency in patients with *PA* infection who were treated with inhaled fluticasone propionate [21]. The interaction between PA and the respiratory mucosa remains inadequately elucidated, with the role of corticosteroids in this process presenting further ambiguity. The observed effectiveness of ICS treatment within this specific patient subgroup implies a potential beneficial role of the interaction between PA or its toxins and the bronchiectatic airways. Given the absence of established treatments for chronic PA infection in the airways of these patients, there is an imperative need to scrutinize the underlying mechanism(s) driving this phenomenon.

Although plausible mechanisms for the beneficial role of ICS in bronchiectasis and airflow obstruction have not been well explored, ICS reportedly suppresses airway inflammation in selected patients with bronchiectasis [30] and COPD [31]. ICS reduced the sputum production and decreased the levels of leucocytes, IL–1b, IL–8, and LTB4 in the sputum [32]. The improvement in the sputum volume is assumed to be the consequence of the downregulation of airway proinflammatory mediators. Decreased inflammatory mediators by ICS could lead to amelioration of leucocyte trafficking, less neutrophilic infiltration, and less release of toxic products into the bronchiectatic airway [33]. In a clinical trial, high-dose ICS reduced the sputum production and improved the symptoms in patients with bronchiectasis [34]. In addition, the complementary mechanism of ICS and LABA may affect the clinical outcomes in bronchiectasis patients. The anti-inflammatory effect of ICS was greater with the concurrent use of beta-agonists through an enhanced translocation of the glucocorticoid receptor or through the potentiated molecular mechanisms of glucocorticoids [35, 36]. In addition, ICS also increased the number of beta-2 receptors or prevented the downregulation of beta-2 receptors by activating gene transcription [36–38]. The synergistic anti-inflammatory effect of ICS/LABAs may overweigh the enhanced bronchodilating effect of LABAs/LAMAs in patients with bronchiectasis and airflow obstruction. Currently, BEC has been considered as a biomarker to identify the subgroup COPD patients who can benefit from ICS treatment. Several post-hoc analyses of clinical trials, utilizing different thresholds for BEC have reported a better response to ICS in patients with a higher baseline BEC [39]. Recent prospective studies have reported a better ICS response for reducing exacerbation in the patients with a higher BEC [40, 41]. Our study suggests that eosinophil can be an important biomarker to predict the response to ICS in the patients with bronchiectasis and airflow obstruction.

There is a paucity of data on the association between inhaled bronchodilators and the clinical course of patients with bronchiectasis and airflow obstruction. Lung function was more improved when bronchiectasis patients were treated with inhaled bronchodilators, especially in patients with a positive bronchodilator response [8, 42, 43]. Adding formoterol to the ICS therapy was related to improved symptoms in the patients with bronchiectasis [7]. A recent RCT showed that tiotropium improved lung function over 6 months in stable patients with bronchiectasis who showed airflow limitations (44). However, benefits in reducing exacerbations or mortality by LABA or LAMA have not been reported in patients with bronchiectasis. In the present study, there were no significant differences in acute exacerbation and lung function decline rate between the ICS/LABA and ICS/LABA/LAMA groups. Considering the greater baseline disease severity of the ICS/LABA/LAMA group compared with ICS/LABA group, there may be a beneficial role of LAMA in patients with bronchiectasis and airflow obstruction.

This study has several limitations. First, our retrospective study analyzed a small number of patients with bronchiectasis and airflow obstruction who used inhaled combination therapy. As ICS tends to be underused in bronchiectasis, our patients are considered to represent a distinct subpopulation of patients with bronchiectasis. Therefore, our results cannot be generalizable to all patients with bronchiectasis. Furthermore, it was challenging to figure out the actual adherence rates or adequate technique rates for inhalers due to the nature of retrospective assessment. More studies with a larger number of patients are needed to generalize the potential benefits of inhaled combination therapy in bronchiectasis and airflow obstruction. Second, it was difficult to distinguish whether the benefit of ICS was related with pathogenesis of bronchiectasis. However, it was found that the benefit of ICS outweighs the potential harm to bronchiectasis in patients with airflow obstruction and eosinophilia. The effect of ICS on eosinophilic inflammation may benefit beyond COPD to bronchiectasis. Third, it was still questionable whether inhaled combination therapy has better clinical outcomes than single inhaled therapy. Because of large clinical heterogeneities, comparisons between single inhaled therapy and inhaled combination therapy cannot be performed properly in retrospective study designs. Instead, we limitedly assumed the additional benefit of ICS while comparing ICS/LABA/LAMA and LABA/LAMA and the additional benefit of LAMA while comparing ICS/LABA/LAMA and ICS/LABA.

## Conclusion

ICS/LABA/LAMA or ICS/LABA may be related with a lower risk of acute exacerbation compared with LABA/LAMA in patients with bronchiectasis and airflow obstruction, especially who had a higher BEC. The annual FEV_1_ decline rate was significantly worsened in the ICS/LABA group compared to the LABA/LAMA group in those with BEC < 200/uL. BEC needs to be further evaluated as a biomarker before the use of ICS in the patients with bronchiectasis and airflow obstruction.

### Electronic supplementary material

Below is the link to the electronic supplementary material.

Supplementary Material 1

## Data Availability

The datasets used and/or analysed during the current study are available from the corresponding author on reasonable request.

## Abbreviations

ABPA: Allergic bronchopulmonary aspergillosis
ACO: Asthma and COPD overlap
BAE: Bronchial artery embolization
BDR: Bronchodilator response
BEC: Blood eosinophil count
BMI: Body mass index
BSI: Bronchiectasis Severity Index
COPD: Chronic obstructive pulmonary disease
CT: Computed tomography
DLCO: Diffusing capacity of the lungs for carbon monoxide
FACED: Forced expiratory volume in 1 s, age, chronic infection with Pseudomonas, radiological extension and dyspnea
FEV1: Forced expiratory volume in 1 s
FVC: Forced vital capacity
GERD: Gastroesophageal reflux disease
hs-CRP: High-sensitivity C-reactive protein
ICS: Inhaled corticosteroid
IQR: Interquartile range
LABA: Long-acting β2-agonist
LAMA: Long-acting muscarinic antagonist
mMRC: Modified Medical Research Council dyspnea scale
NTM–PD: Nontuberculous mycobacteria pulmonary disease
SD: Standard deviation
SE: Standard error
VA: Alveolar volume
