# Supplementary material for: Clinical outcomes of long-term inhaled combination therapies in patients with bronchiectasis and airflow obstruction
Source: BMC Pulm Med. 2024 Jan 23;24:49. doi: 10.1186/s12890-024-02867-4 (PMC10804611; doi:10.1186/s12890-024-02867-4)
Supplement: Supplementary file 1 — Supplementary Material 1 [file 12890_2024_2867_MOESM1_ESM.docx]

**Supplementary information 1. Post-hoc analysis for baseline characteristics according to inhaled combination therapies**

|  | **ICS/LABA/LAMA (n=58)** | **ICS/LABA  (n=52)** | **P-value** | **ICS/LABA/LAMA (n=58)** | **LABA/LAMA  (n=69)** | **P-value** | **ICS/LABA  (n=52)** | **LABA/LAMA  (n=69)** | **P-value** |
| --- | --- | --- | --- | --- | --- | --- | --- | --- | --- |
| **Age, mean (SD)** | 61.05 (10.35) | 62.60 (12.19) | 0.474 | 61.05 (10.35) | 63.81 (11.34) | 0.158 | 62.60 (12.19) | 63.81 (11.34) | 0.573 |
| **Female, n (%)** | 7 (12.1) | 20 (38.5) | 0.001 | 7 (12.1) | 15 (21.7) | 0.151 | 20 (38.5) | 15 (21.7) | 0.045 |
| **BMI, mean (SD)** | 20.44 (4.06) | 22.86 (4.42) | 0.005 | 20.44 (4.06) | 21.86 (3.66) | 0.046 | 22.86 (4.42) | 21.86 (3.66) | 0.195 |
| **Smoking history** |  |  |  |  |  |  |  |  |  |
| Never smoker, n (%) | 10 (17.2) | 20 (38.5) | 0.011 | 10 (17.2) | 25 (36.2) | 0.019 | 20 (38.5) | 25 (36.2) | 0.742 |
| Ex-smoker, n (%) | 25 (43.1) | 18 (34.6) | 0.364 | 25 (43.1) | 29 (42.0) | 0.949 | 18 (34.6) | 29 (42.0) | 0.38 |
| Current smoker, n (%) | 23 (39.7) | 14 (26.9) | 0.158 | 23 (39.7) | 15 (21.7) | 0.031 | 14 (26.9) | 15 (21.7) | 0.527 |
| Pack years, median (IQR) | 30 (22–38) | 15 (9–21) | 0.002 | 30 (22–38) | 20 (14–26) | 0.010 | 15 (9–21) | 20 (14–26) | 0.428 |
| **Disease severity** |  |  |  |  |  |  |  |  |  |
| mMRC score, mean (SD) | 2.09 (0.82) | 1.63 (0.66) | 0.002 | 2.09 (0.82) | 1.75 (0.81) | 0.024 | 1.63 (0.66) | 1.75 (0.81) | 0.389 |
| BSI score, mean (SD) | 7.91 (3.75) | 5.29 (2.80) | <0.001 | 7.91 (3.75) | 6.06 (3.25) | 0.003 | 5.29 (2.80) | 6.06 (3.25) | 0.175 |
| FACED score, mean (SD) | 2.66 (1.66) | 1.63 (1.69) | 0.002 | 2.66 (1.66) | 2.29 (1.48) | 0.192 | 1.63 (1.69) | 2.29 (1.48) | 0.025 |
| **Previous moderate or severe exacerbation history, n (%)** | 24 (43.6) | 14 (29.2) | 0.199 | 24 (43.6) | 14 (20.3) | 0.005 | 14 (29.2) | 14 (20.3) | 0.244 |
| **Bacterial colonizer, n (%)** | 21 (36.2) | 14 (26.9) | 0.297 | 21 (36.2) | 21 (30.4) | 0.694 | 14 (26.9) | 21 (30.4) | 0.492 |
| Pseudomonas colonizer, n (%) | 7 (12.1) | 2 (3.8) | 0.161 | 7 (12.1) | 6 (9.2) | 0.128 | 2 (3.8) | 6 (9.2) | 0.165 |
| **Comorbidity** |  |  |  |  |  |  |  |  |  |
| Hypertension, n (%) | 24 (41.4) | 24 (46.2) | 0.614 | 24 (41.4) | 33 (47.8) | 0.467 | 24 (46.2) | 33 (47.8) | 0.855 |
| Diabetes mellitus, n (%) | 11 (19.0) | 17 (32.7) | 0.099 | 11 (19.0) | 20 (29.0) | 0.19 | 17 (32.7) | 20 (29.0) | 0.661 |
| Chronic kidney disease, n (%) | 6 (10.3) | 3 (5.8) | 0.382 | 6 (10.3) | 6 (8.7) | 0.752 | 3 (5.8) | 6 (8.7) | 0.544 |
| Chronic liver disease, n (%) | 6 (10.3) | 7 (13.5) | 0.613 | 6 (10.3) | 10 (14.5) | 0.483 | 7 (13.5) | 10 (14.5) | 0.872 |
| Cerebrovascular disease, n (%) | 6 (10.3) | 6 (11.5) | 0.841 | 6 (10.3) | 7 (10.1) | 0.97 | 6 (11.5) | 7 (10.1) | 0.806 |
| Cardiovascular disease, n (%) | 9 (15.5) | 13 (25.0) | 0.214 | 9 (15.5) | 14 (20.3) | 0.487 | 13 (25.0) | 14 (20.3) | 0.538 |
| Lung cancer, n (%) | 7 (12.1) | 4 (7.7) | 0.445 | 7 (12.1) | 15 (21.7) | 0.151 | 4 (7.7) | 15 (21.7) | 0.036 |
| Malignancy other than lung cancer, n (%) | 10 (17.2) | 12 (23.1) | 0.445 | 10 (17.2) | 9 (13.0) | 0.509 | 12 (23.1) | 9 (13.0) | 0.149 |
| **Duration of inhaled combination therapy, month, mean (SD)** | 62.81 (39.15) | 55.96 (44.83) | 0.394 | 62.81 (39.15) | 33.34 (17.63) | <0.001 | 55.96 (44.83) | 33.34 (17.63) | 0.001 |

**Note:** Data presented as n (%) or mean (SD) and median (IQR).

**Abbreviations:** ICS, inhaled corticosteroid; LABA, long-acting β2-agonist; LAMA, long-acting muscarinic antagonist; BMI, body mass index; mMRC, Modified Medical Research Council dyspnea scale; BSI, Bronchiectasis Severity Index; FACED, forced expiratory volume in 1 s, age, chronic infection with Pseudomonas, radiological extension and dyspnea; COPD, chronic obstructive pulmonary disease; ACO, Asthma and COPD overlap; NTM–PD, nontuberculous mycobacteria pulmonary disease

**Supplementary information 2. Post-hoc analysis for clinical features according to inhaled combination therapies**

|  | **ICS/LABA/LAMA (n=58)** | **ICS/LABA  (n=52)** | **P-value** | **ICS/LABA/LAMA (n=58)** | **LABA/LAMA  (n=69)** | **P-value** | **ICS/LABA  (n=52)** | **LABA/LAMA  (n=69)** | **P-value** |
| --- | --- | --- | --- | --- | --- | --- | --- | --- | --- |
| **Etiology of bronchiectasis** |  |  |  |  |  |  |  |  |  |
| Post-infectious, n (%) | 28 (48.3) | 14 (26.9) | 0.495 | 28 (48.3) | 29 (42.0) | 0.216 | 14 (26.9) | 29 (42.0) | 0.609 |
| Idiopathic, n (%) | 16 (27.6) | 18 (34.6) | 0.005 | 16 (27.6) | 24 (34.8) | 0.244 | 18 (34.6) | 24 (34.8) | 0.101 |
| Chronic airway disease, n (%) | 9 (15.5) | 10 (19.2) | 0.793 | 9 (15.5) | 8 (11.6) | 0.557 | 10 (19.2) | 8 (11.6) | 0.363 |
| ABPA, n (%) | 2 (3.4) | 5 (9.6) | 0.245 | 2 (3.4) | 3 (4.3) | 0.797 | 5 (9.6) | 3 (4.3) | 0.185 |
| GERD, n (%) | 2 (3.4) | 4 (7.7) | 0.256 | 2 (3.4) | 3 (4.3) | 0.799 | 4 (7.7) | 3 (4.3) | 0.398 |
| Connective tissue disease, n (%) | 0 | 1 (1.9) | 0.84 | 0 | 1 (1.4) | 0.901 | 1 (1.9) | 1 (1.4) | 0.938 |
| Immunosuppression, n (%) | 1 (1.7) | 0 (0.0) | 0.383 | 1 (1.7) | 1 (1.4) | 0.901 | 0 (0.0) | 1 (1.4) | 0.342 |
| **Respiratory symptoms** |  |  |  |  |  |  |  |  |  |
| Cough, n (%) | 25 (43.1) | 23 (44.2) | 0.359 | 25 (43.1) | 25 (36.2) | 0.42 | 23 (44.2) | 25 (36.2) | 0.897 |
| Non-purulent sputum, n (%) | 20 (34.5) | 8 (15.4) | 0.38 | 20 (34.5) | 7 (10.1) | 0.001 | 8 (15.4) | 7 (10.1) | 0.022 |
| Purulent sputum, n (%) | 12 (20.7) | 9 (17.3) | 0.337 | 12 (20.7) | 17 (24.6) | 0.603 | 9 (17.3) | 17 (24.6) | 0.655 |
| Hemoptysis, n (%) | 11 (19.0) | 9 (17.3) | 0.255 | 11 (19.0) | 18 (26.1) | 0.344 | 9 (17.3) | 18 (26.1) | 0.825 |
| Chest discomfort, n (%) | 2 (3.4) | 1 (1.9) | 0.837 | 2 (3.4) | 1 (1.4) | 0.458 | 1 (1.9) | 1 (1.4) | 0.625 |
| Dyspnea, n (%) | 45 (77.6) | 34 (65.4) | 0.928 | 45 (77.6) | 45 (65.2) | 0.113 | 34 (65.4) | 45 (65.2) | 0.113 |
| **Adjuvant treatments** |  |  |  |  |  |  |  |  |  |
| N-acetylcystein, n (%) | 19 (32.8) | 9 (17.3) | 0.841 | 19 (32.8) | 11 (15.9) | 0.026 | 9 (17.3) | 11 (15.9) | 0.063 |
| Ambroxol, n (%) | 19 (32.8) | 11 (21.2) | 0.329 | 19 (32.8) | 20 (29.0) | 0.646 | 11 (21.2) | 20 (29.0) | 0.172 |
| Erdosteine, n (%) | 39 (67.2) | 26 (50.0) | 0.58 | 39 (67.2) | 31 (44.9) | 0.012 | 26 (50.0) | 31 (44.9) | 0.066 |
| Bronchial artery embolization history, n (%) | 9 (15.5) | 4 (7.7) | 0.051 | 9 (15.5) | 14 (20.3) | 0.494 | 4 (7.7) | 14 (20.3) | 0.193 |
| Long–term oxygen therapy, n (%) | 47 (81.0) | 33 (64.7) | 0.288 | 47 (81.0) | 41 (59.4) | 0.235 | 33 (64.7) | 41 (59.4) | 0.042 |
| **Laboratory tests** |  |  |  |  |  |  |  |  |  |
| White blood cell, 1000/uL mean (SD) | 9.54 (13.03) | 7.65 (2.39) | 0.462 | 9.54 (13.03) | 8.03 (2.87) | 0.346 | 7.65 (2.39) | 8.03 (2.87) | 0.309 |
| Hemoglobin, g/dl, mean (SD) | 14.45 (3.69) | 13.46 (1.48) | 0.147 | 14.45 (3.69) | 13.02 (1.74) | 0.005 | 13.46 (1.48) | 13.02 (1.74) | 0.076 |
| Platelet, 1000/ uL mean (SD) | 248 (79) | 266 (87) | 0.223 | 248 (79) | 248 (93) | 0.885 | 266 (87) | 248 (93) | 0.258 |
| Blood eosinophil count, /uL, mean (SD) | 408 (223) | 467 (355) | 0.169 | 408 (223) | 239 (223) | 0.444 | 467 (355) | 239 (223) | 0.062 |
| Blood eosinophil count, n (%) |  |  |  |  |  |  |  |  |  |
| <150/uL | 0 | 0 | - | 0 | 28 (41.2) | <0.001 | 0 | 28 (41.2) | <0.001 |
| 150-299/uL | 20 (34.5) | 22 (42.3) | 0.399 | 20 (34.5) | 23 (33.8) | 0.892 | 22 (42.3) | 23 (33.8) | 0.312 |
| ≥300/uL | 38 (65.5) | 30 (57.7) | 0.399 | 38 (65.5) | 17 (25.0) | <0.001 | 30 (57.7) | 17 (25.0) | <0.001 |
| hs-CRP, median (IQR) | 0.78 (0–2.05) | 0.35 (0–0.70) | <0.001 | 0.78 (0–2.05) | 1.56 (0.89–2.23) | 0.221 | 0.35 (0–0.70) | 1.56 (0.89–2.23) | <0.001 |
| **Spirometric examination** |  |  |  |  |  |  |  |  |  |
| FVC, L, mean (SD) | 2.79 (0.72) | 2.63 (0.98) | 0.209 | 2.79 (0.72) | 2.43 (0.69) | 0.004 | 2.63 (0.98) | 2.43 (0.69) | 0.315 |
| FVC, %, mean (SD) | 76.38 (17.34) | 79.75 (20.02) | 0.009 | 76.38 (17.34) | 70.80 (16.84) | 0.069 | 79.75 (20.02) | 70.80 (16.84) | 0.346 |
| FEV_1_, L, mean (SD) | 1.22 (0.43) | 1.44 (0.61) | 0.01 | 1.22 (0.43) | 1.19 (0.44) | 0.692 | 1.44 (0.61) | 1.19 (0.44) | 0.034 |
| FEV_1_, %, mean (SD) | 47.33 (15.07) | 62.87 (22.85) | 0.001 | 47.33 (15.07) | 49.86 (15.56) | 0.357 | 62.87 (22.85) | 49.86 (15.56) | <0.001 |
| FEV_1_/FVC, %, mean (SD) | 44.13 (12.32) | 56.02 (13.37) | 0.01 | 44.13 (12.32) | 49.66 (12.96) | 0.016 | 56.02 (13.37) | 49.66 (12.96) | <0.001 |
| DLCO, L, mean (SD) | 10.73 (4.88) | 13.10 (5.59) | 0.04 | 10.73 (4.88) | 10.89 (5.16) | 0.879 | 13.10 (5.59) | 10.89 (5.16) | 0.029 |
| DLCO, %, mean (SD) | 63.54 (22.29) | 75.23 (27.99) | 0.136 | 63.54 (22.29) | 66.89 (27.90) | 0.508 | 75.23 (27.99) | 66.89 (27.90) | 0.026 |
| DLCO/VA, L, mean (SD) | 2.92 (1.13) | 3.54 (1.15) | 0.056 | 2.92 (1.13) | 3.08 (1.26) | 0.522 | 3.54 (1.15) | 3.08 (1.26) | 0.009 |
| DLCO/VA, %, mean (SD) | 76.85 (27.87) | 93.25 (28.10) | 0.064 | 76.85 (27.87) | 82.11 (31.59) | 0.378 | 93.25 (28.10) | 82.11 (31.59) | 0.005 |
| BDR positivity, n (%) | 15 (25.9) | 16 (30.8) | 0.002 | 15 (25.9) | 6 (8.8) | 0.011 | 16 (30.8) | 6 (8.8) | 0.568 |
| **Predominant morphology in CT** |  |  |  |  |  |  |  |  |  |
| Cylindrical, n (%) | 27 (46.6) | 22 (42.3) | 0.333 | 27 (46.6) | 22 (31.9) | 0.757 | 22 (42.3) | 22 (31.9) | 0.523 |
| Varicose, n (%) | 17 (29.3) | 18 (34.6) | 0.408 | 17 (29.3) | 29 (42.0) | 0.137 | 18 (34.6) | 29 (42.0) | 0.551 |
| Cystic, n (%) | 14 (24.1) | 12 (23.1) | 0.286 | 14 (24.1) | 18 (26.1) | 0.335 | 12 (23.1) | 18 (26.1) | 0.896 |
| **Total number of lobe involvement in CT, mean (SD)** | 2.64 (1.53) | 2.29 (1.42) | 0.097 | 2.64 (1.53) | 2.75 (1.58) | 0.678 | 2.29 (1.42) | 2.75 (1.58) | 0.219 |

**Note:** Data presented as n (%) or mean (SD) and median (IQR).

**Abbreviations:** ABPA, allergic bronchopulmonary aspergillosis; BAE, bronchial artery embolization; BDR, bronchodilator response; CT, computed tomography; DLCO, diffusing capacity of the lungs for carbon monoxide; FVC, forced vital capacity; FEV_1_, forced expiratory volume in 1 second; GERD, gastroesophageal reflux disease; ICS, inhaled corticosteroid; LABA, long-acting β2-agonist; LAMA, long-acting muscarinic antagonist; SD, standard deviation; SE, standard error; VA, alveolar volume
